# Low Serum Adiponectin May Identify MASLD as a Comorbidity in Patients with Moderate-to-Severe Psoriasis

**DOI:** 10.2147/PTT.S634886

**Published:** 2026-10-03

**Authors:** Luisa Ackermann, Andreas Geier, Matthias Goebeler, Andreas Kerstan, Janik Fleissner

**Affiliations:** 1 Department of Dermatology, Venereology and Allergology, University Hospital Würzburg, Würzburg, Bavaria, Germany; 2 Division of Hepatology, Department of Internal Medicine II, University Hospital Würzburg, Würzburg, Bavaria, Germany

**Keywords:** psoriasis, MASLD, metabolic comorbidity, adiponectin, biomarker

## Abstract

**Purpose:**

Psoriasis is an independent risk factor for metabolic dysfunction-associated steatotic liver disease (MASLD), a condition that frequently remains undetected in clinical routine diagnostics. We investigated whether routine laboratory parameters as well as serum adipokines and cytokines differ between psoriasis patients with and without concurrent MASLD.

**Patients and Methods:**

In this single-center retrospective study, patients with moderate-to-severe plaque psoriasis hospitalized between 2015 and 2024 were analyzed. MASLD was defined as hepatic steatosis (imaging and/or medical records) in conjunction with at least one cardiometabolic risk factor, after exclusion of other liver disease and harmful alcohol use. Serum adipokines and cytokines were quantified by bead-based multiplex assays. Groups were compared by Mann–Whitney U and Fisher exact tests; associations were assessed by Spearman correlation and BMI-adjusted multiple linear regression.

**Results:**

Forty-two psoriasis patients with MASLD (MASLD +) were compared with 17 without (MASLD –). MASLD + patients showed higher BMI (median 32.9 vs 26.7 kg/m^2^, p=0.005), serum ALT (p=0.049), total cholesterol (p=0.007), LDL cholesterol (p=0.023), and CRP (p=0.038), and suffered more frequently from dyslipidemia (p=0.028). Serum adiponectin was significantly lower in MASLD + patients (12.3 vs 22.1 µg/mL, p=0.042), whereas leptin, resistin, adipsin, and all cytokines analyzed did not differ. Adiponectin inversely correlated with BMI (ρ=−0.29, p=0.046); after adjustment for BMI, the group difference was attenuated.

**Conclusion:**

In moderate-to-severe psoriasis, concurrent MASLD is characterized by a metabolically adverse profile and reduced serum adiponectin. As the difference is partly explained by BMI, adiponectin warrants prospective evaluation as a candidate marker of concurrent MASLD.

## Introduction

Metabolic dysfunction-associated steatotic liver disease (MASLD, formerly known as non-alcoholic fatty liver disease) has become the second leading cause of chronic liver disease with a global prevalence of 30%.^1^,^2^ It comprises a spectrum ranging from steatosis to metabolic dysfunction-associated steatohepatitis (MASH), which can progress to fibrosis, cirrhosis, and hepatocellular carcinoma.^3^ MASLD is diagnosed when hepatic steatosis of no other identifiable cause coexists with at least one cardiometabolic risk factor (CMRF).^4^ CMRFs are well established criteria for the diagnosis of metabolic syndrome and comprise abdominal obesity, elevated serum triglyceride levels, reduced high-density lipoprotein cholesterol levels, elevated blood pressure, and elevated fasting glucose.^5^

Psoriasis is an immune-mediated inflammatory skin and joint disease associated with metabolic syndrome.^6^,^7^ In line with this cardiometabolic risk profile, MASLD is present in approximately 50% of psoriasis patients and thus substantially more frequent than in non-psoriatic individuals.^8^,^9^ Conversely, MASLD itself is closely linked to an increased risk of cardiovascular disease.^10^ Beyond these metabolic associations, both psoriasis and MASLD share an interleukin-17 (IL-17)-driven pathogenesis.^11–15^ Despite this frequent coexistence, asymptomatic MASLD may remain undetected in patients with psoriasis due to insufficient screening and limited sensitivity of non-invasive clinical MASLD scores.^7^,^9^,^16^ Although widely used, abdominal ultrasound has limited sensitivity for detecting mild steatosis and does not allow for reliable assessment of hepatic fibrosis. Therefore, the current MASLD guideline recommends a stepwise approach based on non-invasive scores and elastography.^16^ To date, no biomarker is available that reliably identifies MASLD comorbidity in psoriasis patients. While numerous monoclonal antibodies targeting pro-inflammatory cytokine signaling have been approved for the treatment of moderate-to-severe psoriasis,^17–28^ no IL-17-based therapeutic concept has been established for MASLD treatment. Resmetirom, a thyroid hormone receptor β-selective agonist, and semaglutide, a glucagon-like peptide-1 receptor agonist, are currently the only agents approved for the treatment of noncirrhotic MASH with moderate-to-advanced fibrosis.^29^,^30^ Interestingly, a growing body of evidence suggests that IL-17- and IL-23-targeting biologics may exert favorable hepatic effects by decreasing serum aminotransferases and non-invasive fibrosis indices.^31–33^ Besides cytokines, adipose-tissue-derived adipokines such as adiponectin, leptin, resistin, and adipsin provide a possible mechanistic link between psoriasis and MASLD. In mice with imiquimod-induced psoriasiform dermatitis, adiponectin was shown to exert anti-inflammatory effects by directly suppressing IL-17 production from dermal γδT cells via the AdipoR1 receptor and adiponectin-deficient mice consequently developed a more pronounced skin inflammation.^34^ In line, adiponectin was reported to reduce IL-17 secretion from human CD4^+^ and CD8^+^ T cells.^34^ This is consistent with a meta-analysis of observational studies that demonstrate lower serum adiponectin concentrations in psoriasis patients as compared to controls,^35^ and a case-control study reporting reduced serum adiponectin concentrations independent of metabolic syndrome.^36^ However, an earlier meta-analysis found no significant overall difference in adiponectin between psoriasis patients and controls.^37^ In patients with MASLD, circulating adiponectin was shown to decline with progression to MASH and fibrosis.^38^ Therefore, reduced serum adiponectin appears to be a shared pathophysiological feature of both psoriasis and MASLD, which supports its evaluation as a potential biomarker for MASLD comorbidity in psoriasis patients.

Of note, hypercholesterolemia is common among patients with both psoriasis and MASLD and is often treated with statins. Besides lipid lowering effects, statins were shown to reduce serum levels of pro-inflammatory cytokines IL-6 and tumor necrosis factor (TNF)-α.^39^ In a meta-analysis of 43 randomized trials, rosuvastatin had a significant adiponectin-lowering effect by 0.7 µg/mL while pitavastatin significantly increased the serum adiponectin concentration by 0.5 μg/mL; atorvastatin, simvastatin, and pravastatin had numerically increasing but non-significant effects.^40^ Intake of statins has been linked to short-term PASI improvement in psoriasis at week eight of treatment and appears to be both safe and potentially beneficial in MASLD.^41^,^42^

In the present study, we compared clinical characteristics and serum concentrations of adipokines, cytokines, and routine laboratory parameters between psoriasis patients with and without MASLD, to examine whether adiponectin can help identify concurrent MASLD.

## Methods

### Study Design and Study Population

This retrospective study was conducted at the Department of Dermatology, Venereology and Allergology, University Hospital Würzburg, Germany. Clinical data and serum samples were collected at the time of hospital admission from patients with moderate-to-severe plaque-type psoriasis (Psoriasis Area and Severity Index, PASI > 10, indicative of relevant systemic inflammation), who were hospitalized between 2015 and 2024, and off systemic treatment at sampling (conventional systemic treatment for > 30 days; biologics for > 3 months). In case of relapsing disease, patients were only included once. Patients with a history of viral hepatitis, concomitant hepatotoxic medication, or self-reported harmful alcohol consumption were excluded from the analysis. In addition, 28 patients whose liver status had not been assessed could not be assigned to either group and were thus excluded from the analysis. After these exclusions, 59 patients with psoriasis were available for analysis and were grouped according to MASLD comorbidity into MASLD-positive (MASLD +) and MASLD-negative (MASLD −) (Figure 1). In accordance with current consensus criteria, MASLD was diagnosed based on hepatic steatosis as documented by abdominal ultrasound and/or medical records, together with at least one CMRF.^4^,^16^ CMRFs (overweight/obesity, arterial hypertension, type 2 diabetes mellitus, and dyslipidemia) were assessed from routine diagnostics or medical records, including lipid-lowering, antihypertensive, and antidiabetic medication. Information on medication was obtained from the electronic health records.

**Figure 1:**
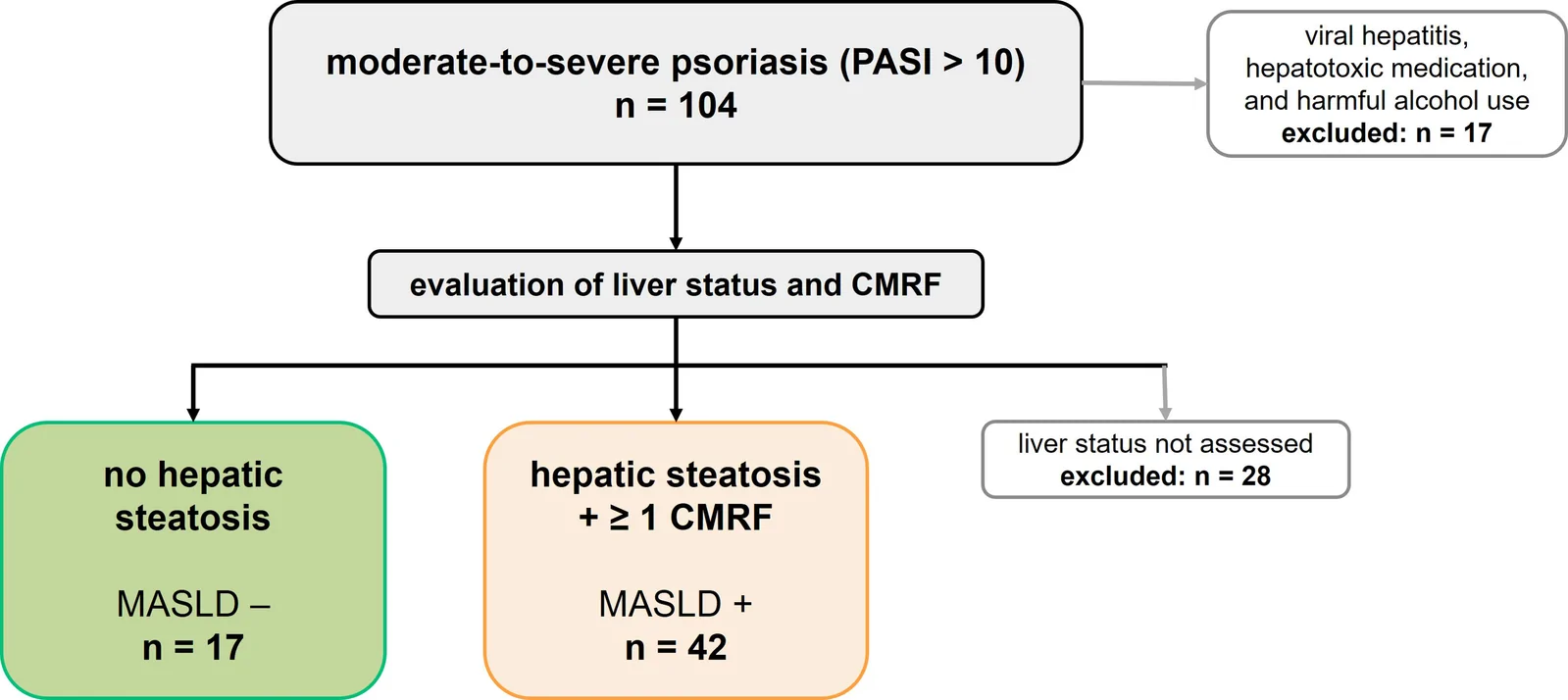
Patient selection. One hundred four patients with moderate-to-severe psoriasis (PASI > 10) were screened for inclusion. MASLD was defined as hepatic steatosis, documented by imaging or medical records, together with at least one cardiometabolic risk factor (CMRF), after exclusion of other liver disease and harmful alcohol use. Forty-two patients with (MASLD +) and 17 without (MASLD −) MASLD were identified. Since liver status could not be assessed in 28 patients, these were thus excluded from the analysis, as these patients could not be assigned to either group.

**Abbreviations**: CMRF, cardiometabolic risk factor; MASLD –, psoriasis without metabolic dysfunction-associated steatotic liver disease; MASLD +, psoriasis with metabolic dysfunction-associated steatotic liver disease; PASI, Psoriasis Area and Severity Index.

The following variables from the routine diagnostic work-up were considered: age, sex, body mass index (BMI), PASI, Dermatology Life Quality Index (DLQI), aspartate aminotransferase (AST), alanine aminotransferase (ALT), γ-glutamyl transferase (γ-GT), platelet count, neutrophil count, lymphocyte count, serum triglycerides, total cholesterol including high- and low-density lipoprotein (HDL and LDL) cholesterol, C-reactive protein (CRP), and systemic treatment. The neutrophil-to-lymphocyte ratio (NLR) was calculated as the ratio of neutrophil to lymphocyte counts. The non-invasive Fibrosis-4 index (FIB-4) was calculated as: $\left({{\mathrm{age }}\left[{{\mathrm{years}}} \right]{\mathrm{ \times\ AST }}\left[{{\mathrm{U/L}}} \right]} \right){\mathrm{/(platelet\ count }}\left[{{\mathrm{ \times 1}}{{\mathrm{0}}^{\mathrm{9}}}{\mathrm{/L}}} \right]{\mathrm{ \times }}\sqrt {{\mathrm{ALT}}} \,\left[{{\mathrm{U/L}}} \right]{\mathrm{)}}$.^43^

### Serum Collection and Bead-Based Multiplex Assay

Peripheral venous whole blood was collected in serum tubes and centrifuged at 760 g for 5 minutes at room temperature. The serum was aliquoted and stored at −80 °C until analysis. Serum concentrations of adipokines (adiponectin, leptin, resistin, adipsin) and cytokines (IL-1β, IFN-α2, IFN-γ, TNF-α, CCL2/MCP-1, IL-6, IL-8, IL-10, IL-12p70, IL-17A, IL-18, IL-23, IL-33) were determined in all 59 patients with known liver status using the LEGENDplex Human Metabolic Panel 1 and the Human Inflammation Panel (BioLegend) according to the manufacturer’s instructions. Data were acquired on a CytoFLEX LX flow cytometer (Beckman Coulter) and analyzed with LEGENDplex software, with concentrations interpolated from standard curves.

### Statistical Analysis

Continuous variables are presented as median [interquartile range] and were compared using the Mann–Whitney *U*-test as normal distribution was not assumed given the limited sample size. P-values were adjusted for multiple testing within the adipokine and cytokine panels separately by using the Benjamini-Hochberg false-discovery-rate method. Categorical variables are presented as counts (percentages) and compared using the Fisher exact test. Associations of serum adipokines with BMI and routine laboratory parameters were assessed using the Spearman rank correlation coefficient. Similarly, correlations of psoriatic disease activity, reflected by Psoriasis Area and Severity Index (PASI), Dermatology Life Quality Index (DLQI), and NLR, with hepatic and metabolic parameters were assessed. Group differences were re-tested after adjustment for BMI by multiple linear regression. A two-sided p-value < 0.05 was considered statistically significant. Analyses were performed in GraphPad Prism v10.

## Results

### Patient Characteristics and Routine Laboratory Parameters

Forty-two (71.2%) of the patients with moderate-to-severe psoriasis and available liver status fulfilled the criteria for MASLD (MASLD +) and 17 (28.8%) had no hepatic steatosis (MASLD −). In 28 patients, liver status had not been assessed, and these patients were therefore excluded (Figure 1). The two groups were well balanced regarding age (median 55 [44–61] vs 49 [41–62] years, p = 0.55), sex (67% vs 76% male, p = 0.55), psoriasis burden (PASI 13.8 vs 15.5, p = 0.40), quality of life (DLQI 18.5 vs 17.5, p = 0.92), non-biologic systemic therapy (19% vs 35%, p = 0.197), and biologic therapy (29% vs 12%, p = 0.310) (Table 1).

**Table 1:** Clinical and Laboratory Characteristics

Variable	MASLD – (n=17)	MASLD + (n=42)	p
**Demographics**			
Age (years)	49.0 [41.0–62.0]	55.0 [44.2–60.8]	0.546
Male sex, n (%)	13 (76%)	28 (67%)	0.545
BMI (kg/m^2^)	26.7 [22.4–28.8]	32.9 [27.3–38.7]	0.005 (**)
**Psoriasis severity**			
PASI	15.5 [11.6–22.2]	13.8 [11.7–18.6]	0.399
DLQI	17.5 [14.0-24.0]	18.5 [14.0–21.5]	0.924
**Cardiometabolic risk factors, n (%)**			
Arterial hypertension	6 (35%)	22 (54%)	0.255
Type 2 diabetes	3 (19%)	11 (27%)	0.735
Dyslipidemia	3 (21%)	18 (58%)	0.028 (*)
**Previous systemic therapy, n (%)**			
None (treatment-naïve)	9 (53%)	22 (52%)	1.000
Non-biologic^†^	6 (35%)	8 (19%)	0.197
Biologic^‡^	2 (12%)	12 (29%)	0.310
**Liver enzymes**			
AST (U/L)	25.3 [20.8–38.3]	30.0 [24.0–36.4]	0.274
ALT (U/L)	24.6 [15.5–39.8]	37.6 [25.5–54.0]	0.049 (*)
γ-GT (U/L)	29.7 [16.4–48.1]	49.1 [25.6–75.5]	0.068
**Lipids and cholesterol-lowering therapy, n (%)**			
Total cholesterol (mg/dL)	152 [126–170]	206 [176–231]	0.007 (**)
Triglycerides (mg/dL)	100 [93–136]	151 [106–209]	0.082
HDL-C (mg/dL)	51 [45–58]	44 [33–56]	0.270
LDL-C (mg/dL)	74 [64–92]	128 [95–145]	0.023 (*)
**Cholesterol-lowering therapy, n (%)**	2^#^ (11.8%)	4° (9.5%)	**1.000**
**Other**			
CRP (mg/dL)	0.16 [0.09–0.34]	0.44 [0.24–0.98]	0.038 (*)
Platelets (per nL)	258 [215–308]	261 [223–313]	0.967
FIB-4	1.16 [0.79–1.32]	0.95 [0.68–1.31]	0.838
NLR	2.80 [2.46–4.11]	3.22 [2.44–4.36]	0.744

**Notes**: Data are shown as median [interquartile range] or n (%). Mann–Whitney *U*-test for continuous variables; Fisher exact test for categorical variables. P-values in bold indicate significance, p < 0.05 (*) and p < 0.01 (**), respectively. Serum was obtained after a treatment-free interval with washout of at least four weeks (non-biologic) or at least three months (biologic). ^†^Previous systemic treatment with dimethyl fumarate, methotrexate, or apremilast; ^‡^previous systemic treatment with TNF (adalimumab), IL-17 (secukinumab), IL-12/23 (ustekinumab), and IL-23 (guselkumab, tildrakizumab) inhibitors (no significant difference between groups); ^#^both patients received simvastatin; °three patients received simvastatin and one patient received atorvastatin.

**Abbreviations**: ALT, alanine aminotransferase; AST, aspartate aminotransferase; BMI, body mass index; CRP, C-reactive protein; DLQI, Dermatology Life Quality Index; FIB-4, fibrosis-4 index; γ-GT, γ-glutamyl transferase; HDL-C, high-density lipoprotein cholesterol; LDL-C, low-density lipoprotein cholesterol; MASLD –, psoriasis without metabolic dysfunction-associated steatotic liver disease; MASLD +, psoriasis with metabolic dysfunction-associated steatotic liver disease; NLR, neutrophil-to-lymphocyte ratio; PASI, Psoriasis Area and Severity Index.

MASLD + patients were significantly more obese, with a higher median BMI (32.9 [27.3–38.7] vs 26.7 [22.4–28.8] kg/m^2^, p = 0.005). Dyslipidemia was significantly more prevalent in MASLD + than in MASLD − patients (58% vs 21%, p = 0.028), whereas arterial hypertension (54% vs 35%, p = 0.255) and type 2 diabetes (27% vs 19%, p = 0.735) did not differ between groups (Table 1). Consistent with MASLD, serum ALT was significantly higher in MASLD + patients (37.6 [25.5–54.0] vs 24.6 [15.5–39.8] U/L, p = 0.049), with a similar trend for γ-GT (49.1 [25.6–75.5] vs 29.7 [16.4–48.1] U/L, p = 0.068). AST did not differ between groups (p = 0.27). The non-invasive fibrosis score FIB-4 (0.95 [0.68–1.31] vs 1.16 [0.79–1.32], p = 0.84) did not distinguish MASLD status, underscoring the limited sensitivity of FIB-4 for steatosis in the absence of advanced fibrosis (Table 1). The neutrophil-to-lymphocyte ratio (NLR) did not differ between groups (3.22 [2.44–4.36] vs 2.80 [2.46–4.11], p = 0.74). MASLD + displayed an atherogenic lipid profile, with significantly higher total cholesterol (206 [176–231] vs 152 [126–170] mg/dL, p = 0.007) and LDL cholesterol (128 [95–145] vs 74 [64–92] mg/dL, p = 0.023), a trend toward higher triglycerides (151 [106–209] vs 100 [93–136] mg/dL, p = 0.08), and numerically lower HDL cholesterol (44 [33–56] vs 51 [45–58] mg/dL, p = 0.27). Statins were used in only 2/17 MASLD – patients and 4/42 MASLD + patients. None of the patients received rosuvastatin or pitavastatin (Table 1). Systemic low-grade inflammation, reflected by CRP, was also higher in the MASLD group (0.44 [0.24–0.98] vs 0.16 [0.09–0.34] mg/dL, p = 0.038) (Table 1 and Supplementary Figure S1).

### Serum Cytokine and Adipokine Profiles

Serum adiponectin was the only adipokine that differed significantly between groups and was lower in MASLD + patients (12.3 [10.5–17.8] vs 22.1 [17.4–27.0] µg/mL, p = 0.042); however, after Benjamini-Hochberg correction for multiple comparisons, this difference did not remain statistically significant (q = 0.17). Leptin (8.4 [4.7–22.5] vs 8.4 [4.3–13.3] ng/mL, p = 0.74), resistin (1.47 [0.87–3.38] vs 1.50 [1.13–2.92] ng/mL, p = 0.57), and adipsin (1032 [803–1312] vs 1191 [652–1862] ng/mL, p = 0.61) did not differ between groups (Supplementary Table S1 and Figure 2). None of the 13 measured cytokines differed significantly between patients with and without MASLD, either before or after FDR correction (Supplementary Table S2 and Supplementary Figure S2). Unexpectedly, several pro-inflammatory cytokines, including CCL2 (p = 0.09) and IL-8 (p = 0.08), showed a non-significant trend toward lower serum concentrations in MASLD + patients.

**Figure 2:**
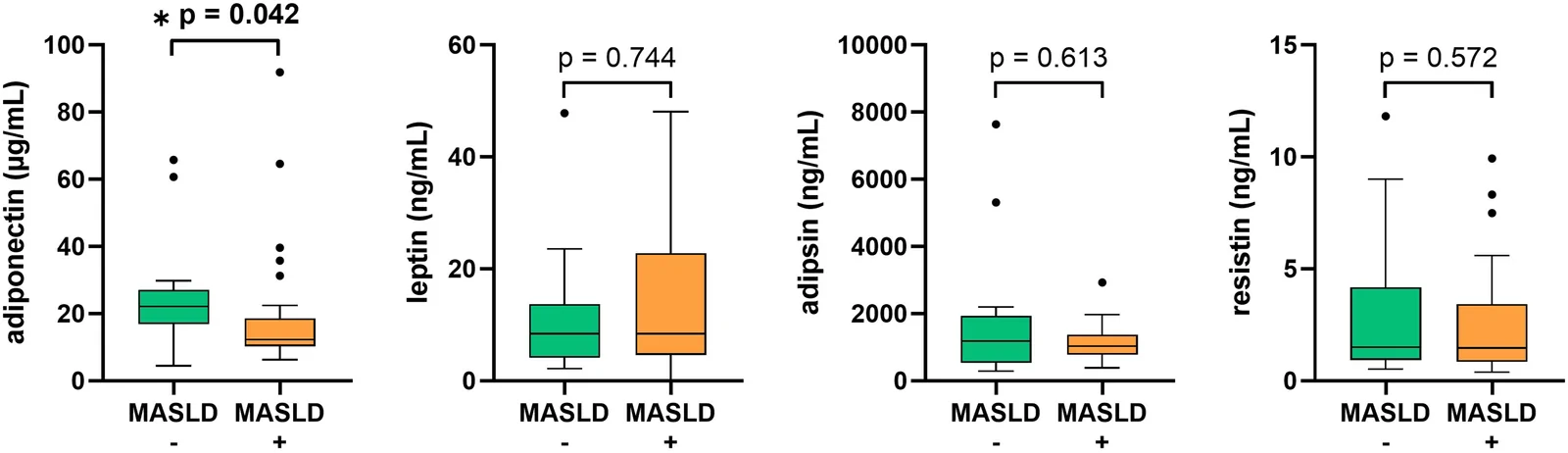
Serum adipokine concentrations. Serum adipokine concentrations were assessed by bead-based multiplex assay in psoriasis patients with and without concurrent MASLD: adiponectin, leptin, adipsin, and resistin. Box-and-whisker plots show the median as horizontal line and the interquartile range as box; whiskers extend to 1.5 x the interquartile range (Tukey), and the value outside this range is plotted as an individual point. Groups were compared using the Mann–Whitney *U*-test; significant differences are indicated as *p < 0.05.

**Abbreviations**: MASLD –, psoriasis without metabolic dysfunction-associated steatotic liver disease; MASLD +, psoriasis with metabolic dysfunction-associated steatotic liver disease.

### Correlation Analysis of Adipokines and BMI

Across the whole psoriasis cohort, serum leptin concentrations correlated positively with BMI (Spearman’s ρ = 0.46, p = 0.001; Figure 3A), whereas adiponectin concentrations showed a weaker inverse correlation with BMI (ρ = −0.29, p = 0.046; Figure 3B). None of the measured adipokines correlated significantly with liver enzymes or lipid parameters (Supplementary Table S3). In addition, we did not observe significant correlations between markers of psoriatic disease severity (PASI, DLQI, NLR) and hepatic or metabolic parameters (Supplementary Table S4).

**Figure 3:**
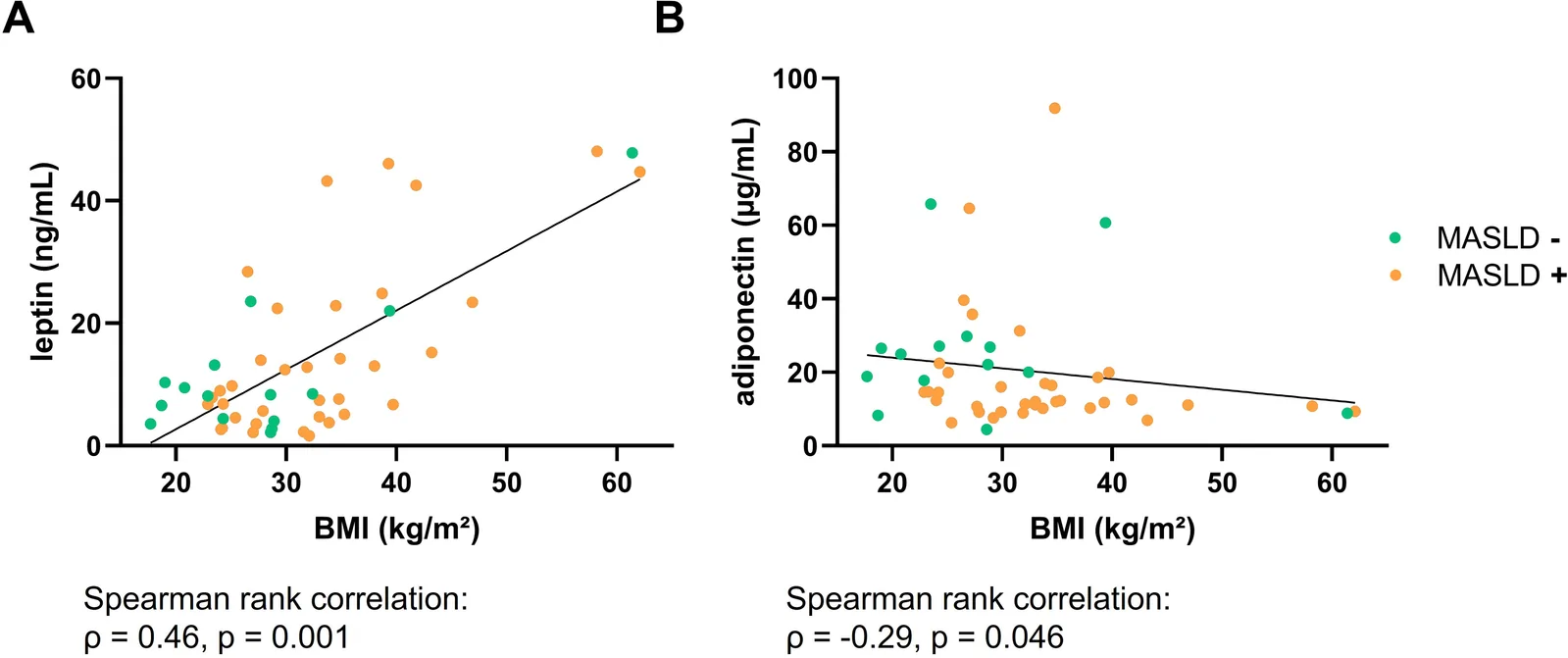
Correlation of serum adiponectin and leptin with BMI. Scatter plots of serum leptin (**A**) and adiponectin (**B**) versus BMI with pooled log-linear regression lines. The association of each adipokine with BMI was tested by Spearman rank correlation (correlation coefficient *ρ* and two-tailed p-value shown).

**Abbreviations**: BMI, body mass index; MASLD –, psoriasis without metabolic dysfunction-associated steatotic liver disease; MASLD +, psoriasis with metabolic dysfunction-associated steatotic liver disease.

Because adiponectin concentrations were significantly lower in MASLD + patients, we assessed whether this difference was confounded by BMI. After BMI adjustment, the reduction in adiponectin concentrations in MASLD + patients was attenuated and no longer reached statistical significance (BMI-adjusted p = 0.13), although the direction of the effect was preserved (Supplementary Figure S3A). Leptin did not differ between groups either before or after BMI adjustment (BMI-adjusted p = 0.71, Supplementary Figure S3B), consistent with leptin concentrations being primarily associated with BMI in this cohort.

## Discussion

In this retrospective cohort of patients with moderate-to-severe psoriasis, concurrent MASLD was associated with an adverse metabolic profile, including obesity, elevated serum ALT, CRP, and cholesterol, and a significantly reduced serum adiponectin concentration. In contrast, leptin, resistin, adipsin, and a broad panel of circulating cytokines did not discriminate between MASLD + and MASLD − patients. These findings are in line with earlier observations made when the NAFLD terminology was still in use^8^ and are consistent with comparative studies demonstrating high concordance between the former NAFLD and the current MASLD definitions.^44^,^45^ By investigating this clinically important psoriasis comorbidity our study extends previous observations and identifies adiponectin as a candidate biomarker.

Notably, concurrent MASLD was present in more than 70% of patients, exceeding previously reported frequencies.^8^,^46^,^47^ This overrepresentation may reflect selection bias, as patients with a higher metabolic disease burden are more likely to be hospitalized. In addition, the 28 patients without liver status assessment may have been less likely to have MASLD, as hepatic screening was rather omitted in patients who were lean, younger, or without cardiometabolic risk factors.

In contrast to the prominent role of IL-17 in inflamed skin and liver tissue, we did not detect higher circulating cytokine concentrations in MASLD + patients. This might implicate a specific skin-liver axis that is missed when solely relying on serum cytokine levels rather than on local concentrations at the sites of inflammation. Systemic therapy exposure was balanced between the groups, making a major confounding effect of ongoing anti-inflammatory treatment on the measured biomarkers unlikely.

Adiponectin is an anti-inflammatory adipokine whose serum concentrations decrease with increasing obesity.^48^,^49^ The lower adiponectin concentrations observed in our MASLD + patients are biologically plausible and reflected in various experimental animal models.^50–54^ The inverse correlation between adiponectin and BMI in our cohort indicates that obesity is a confounder that contributes to, but does not fully explain, the lower adiponectin concentrations in MASLD + patients. Interestingly, despite its strong coupling to BMI (ρ = 0.46, p < 0.001), leptin did not differ between MASLD − and MASLD + patients, which may suggest that low adiponectin does not mirror obesity alone, although this observation is exploratory and requires confirmation. Nonetheless, BMI accounted for a substantial part of the difference, which lost statistical significance after adjustment. Our data thus indicate that adiponectin is associated with obesity, which is linked to MASLD, rather than reflecting a BMI-independent biomarker of MASLD. Future studies should therefore validate adiponectin as a biomarker in larger cohorts to determine whether its association with MASLD is truly independent of BMI.

Identifying psoriasis patients with concurrent MASLD has direct clinical implications, because MASLD frequently remains undiagnosed and affected patients are at increased cardiovascular risk.^10^,^55^ A biomarker such as adiponectin, interpreted together with BMI and routine clinical and laboratory parameters, could complement existing non-invasive MASLD scores in patients with psoriasis. While serum adiponectin differed significantly between groups, this finding is of exploratory nature and does not reflect established diagnostic accuracy.

This study has several limitations. Its retrospective, single-center design, modest sample size and imbalanced group sizes limit statistical power. MASLD was defined using imaging and medical records rather than liver biopsy, and liver status could not be assessed in a subset of patients. As ultrasound has limited sensitivity for detecting mild steatosis and fibrosis, some patients may have been misclassified as MASLD –. However, this would likely have attenuated the difference in serum adiponectin levels between the two groups. The difference in adiponectin was of moderate statistical significance and partly confounded by BMI. Although statins have been reported to modulate serum adipokine and cytokine levels, only a single-digit number of patients in our study were taking statins. Therefore, the potential for confounding by statin treatment in our analysis is likely to be limited. Larger, prospective multicenter studies with biopsy- or elastography-confirmed MASLD and multivariable adjustment are needed to confirm the discriminatory value of adiponectin.

## Conclusion

In patients with moderate-to-severe psoriasis, concurrent MASLD is characterized by a metabolically detrimental phenotype and by significantly reduced serum adiponectin, whereas other adipokines and a broad cytokine panel do not discriminate between groups. These data support further evaluation of adiponectin as a candidate biomarker that warrants further BMI-adjusted validation in larger cohorts to identify psoriasis patients at risk of concurrent MASLD.

## Data Availability

The data that support the findings of this study are available from the corresponding author upon reasonable request.

## Abbreviations

ALT, alanine aminotransferase; AST, aspartate aminotransferase; BMI, body mass index; CCL2, C-C motif chemokine ligand 2; CMRF, cardiometabolic risk factor; CRP, C-reactive protein; DLQI, Dermatology Life Quality Index; FIB-4, Fibrosis-4 index; γ-GT, γ-glutamyl transferase; HDL, high-density lipoprotein; IFN, interferon; IL, interleukin; IQR, interquartile range; LDL, low-density lipoprotein; MASH, metabolic dysfunction-associated steatohepatitis; MASLD, metabolic dysfunction-associated steatotic liver disease; MASLD +, psoriasis with concurrent MASLD; MASLD −, psoriasis without MASLD; NAFLD, non-alcoholic fatty liver disease; NLR, neutrophil-to-lymphocyte ratio; PASI, Psoriasis Area and Severity Index; TNF-α, tumor necrosis factor-α.
